# Mental Representations of Time in English Monolinguals, Mandarin Monolinguals, and Mandarin–English Bilinguals

**DOI:** 10.3389/fpsyg.2022.791197

**Published:** 2022-02-10

**Authors:** Wenxing Yang, Yiting Gu, Ying Fang, Ying Sun

**Affiliations:** College of Foreign Studies, Yangzhou University, Yangzhou, China

**Keywords:** English, Mandarin, monolingual, bilingual, L2 proficiency, mental representations of time

## Abstract

This study recruited English monolinguals, Mandarin monolinguals, and Mandarin–English (ME) bilinguals to examine whether native English and native Mandarin speakers think about time differently and whether the acquisition of L2 English could reshape native Mandarin speakers’ mental representations of temporal sequence. Across two experiments, we used the temporal congruency categorization paradigm which involved two-alternative forced-choice reaction time tasks to contrast experimental conditions that were assumed to be either compatible or incompatible with the internal spatiotemporal associations. Results add to previous studies by confirming that native English and native Mandarin speakers do think about time differently, and the significant crosslinguistic discrepancy primarily lies in the vertical representations of time flow. However, current findings also clarify the existing literature, demonstrating that the acquisition of L2 English does not appear to affect native Mandarin speakers’ temporal cognition. ME bilinguals, irrespective of whether they attained elementary or advanced level of English proficiency, exhibited temporal thinking patterns commensurate with those of Mandarin monolinguals. Some theoretical implications regarding the effect of bilingualism on cognition in general can be drawn from the present study, a crucial one being that it provides evidence against the view that L2 acquisition can reshape habitual modes of thinking established by L1.

## Introduction

Theoretical issues concerning the language–thought relationship in general and the effect of bilingualism on cognition in particular have gained due respect in the past two decades (e.g., Jarvis and Pavlenko, 2008; Kousta et al., 2008; Athanasopoulos, 2009; Han and Cadierno, 2010; Athanasopoulos et al., 2011; Park and Ziegler, 2014; Flecken et al., 2015; Gerwien and von Stutterheim, 2018). According to linguistic relativity or Sapir–Whorf hypothesis (Whorf, 1956), a person’s native language may serve as one of the primary sources that influence the way through which a person thinks about the world and crosslinguistic differences may bring about distinctions in how people of different languages conceptualize the world (Whorf, 1956). Although non-verbal cognition does not always correspond to linguistic expressions in all cognitive domains, the Whorfian effects were found at least in certain domains, such as color, motion, and space (e.g., Levinson, 1996; Roberson, 2005; Han and Cadierno, 2010; Hendriks and Hickmann, 2011; Flecken et al., 2014; Cibelli et al., 2016; Gerwien and von Stutterheim, 2018). If speakers of different languages differ in their thinking patterns, does learning novel lexical and grammatical categories in the second language (L2) restructure cognition, or is cognition fixed once-and-for-all by L1 (first language) to which a person is exposed? Inspired by linguistic relativity hypothesis, the theoretical positions, and experimental findings from studies available on bilingualism and thought, researchers now take seriously the possibility that bilinguals might have a different worldview from monolinguals as a result of using languages with contrasting linguistic categories (e.g., Jarvis and Pavlenko, 2008; Han and Cadierno, 2010; Bassetti and Cook, 2011; Pavlenko, 2011). In other words, additional language learning may have the power of transforming speakers’ cognition, especially when there are significant disparities between L1 and L2.

### English and Mandarin Speakers’ Mental Representations of Time

Among the forgoing investigations of the link between monolingualism/bilingualism and thought, a line of accumulating research explored English and Mandarin speakers’ mental representations of time. Relevant questions covered by the existing literature included, but were not limited to how English and Mandarin speakers’ space–time mappings were affected by cultural conventions, linguistic factors, bodily experience, etc. (e.g., Boroditsky, 2001; Chen, 2007; Boroditsky et al., 2011; Miles et al., 2011; Lai and Boroditsky, 2013; Li and Cao, 2017; Gu et al., 2018; Hendricks et al., 2018; Li et al., 2019). Across these studies, the relationship between metaphoric language and temporal cognition (e.g., Boroditsky, 2001; Chan and Bergen, 2005; Chen, 2007; Boroditsky et al., 2011; Fuhrman et al., 2011; Miles et al., 2011; Chen and O’Seaghdha, 2013; Lai and Boroditsky, 2013; Yang and Sun, 2016a,b) has attracted much attention and controversy. People around the world may rely on space to talk about time, but the particular mappings from space to time vary across languages. English predominately employs sagittal front/back spatiotemporal metaphors to depict time (e.g., Wednesday comes *before* Thursday, past events lie *behind* us). In contrast, Mandarin systematically adopts both sagittal (*qián*/front and *hòu*/back) and vertical top/bottom expressions (*shàng* and *xià*). In Mandarin vertical spatiotemporal metaphors, *Shàng* (“up”) refers to an earlier event or time point, for example, *shàng xīng qī* (“last week”), and *xià* (“down”) refers to a later event or time point, for example., *xià gè yuè* (“next month”). Given the crosslinguistic differences between English and Mandarin in patterns of spatiotemporal metaphors, English and Mandarin speakers are supposed to differ in their mental representations of time. Several experimental paradigms were used to test such a hypothesis, which included: (a) the priming paradigm which examined the effects of processing spatial primes on the understanding of temporal targets (Boroditsky, 2001; Chen, 2007); (b) the temporal congruency categorization paradigm which involved two-alternative forced-choice reaction time tasks to contrast experimental conditions that were either compatible or incompatible with the assumed spatiotemporal associations (Boroditsky et al., 2011; Fuhrman et al., 2011; Miles et al., 2011); (c) the graphic output of temporal order paradigm which asked participants to arrange in order a series of cards which depict temporal sequences (Chan and Bergen, 2005; Yang and Sun, 2016b); (d) the gesture paradigm which elicited participants’ spontaneous co-speech gestures about temporal relations (Casasanto and Jasmin, 2012; Gu et al., 2019). Some of the studies found that English speakers conceive of time as sagittal-oriented, whereas Mandarin speakers display a salient tendency to represent time vertically (e.g., Boroditsky, 2001; Chan and Bergen, 2005). In other studies, English speakers were shown to access sagittal temporal reasoning while Mandarin speakers rely on both a sagittal and a vertical line to conceptualize elapsing time (e.g., Boroditsky et al., 2011; Fuhrman et al., 2011; Yang and Sun, 2016a). Two studies (Grace and Vogel, 2008; Liu and Zhang, 2009) even claimed that Mandarin speakers may possess a single vertical mental timeline (MTL). Irrespective of the diversity in experimental paradigms, converging conclusions drawn from much of the empirical work is that English and Mandarin speakers do think about time differently.

On the basis of the observed discrepancies between English and Mandarin speakers, a few researches further probed into the possible influence of L2 English on habitual temporal thinking patterns of Mandarin–English (ME) bilinguals (Boroditsky, 2001; Fuhrman et al., 2011; Miles et al., 2011; Lai and Boroditsky, 2013). Preliminary findings revealed that the acquisition of L2 English spatiotemporal metaphors reshaped Mandarin speakers’ mental representations of time. Factors, such as earlier age of L2 acquisition and longer duration of residence in English-speaking countries, were documented to attenuate ME bilinguals’ significant vertical bias in their temporal cognition (Boroditsky, 2001; Fuhrman et al., 2011). In the meantime, Miles et al. (2011) found that ME bilinguals accommodated both a sagittal and a vertical timeline in their minds. The sagittal space–time mapping was interpreted to be entirely constructed by linguistic forces of L2 English and the vertical representation commensurate with L1 Mandarin.^1^ The diminished magnitude of the vertical bias in thinking about time, together with the identification of a sagittal MTL, was taken as evidence of a robust impact of L2 English on restructuring Mandarin speakers’ temporal cognition. For one thing, English almost exclusively use sagittal spatiotemporal metaphors to express temporal relations. For another, ME bilinguals were claimed to diverge from native speakers of Mandarin but resemble native speakers of English in their mental representations of time, given their performances in the experiments (Fuhrman et al., 2011; Miles et al., 2011).

Some potential limitations may undermine the abovementioned results. The first issue points to the selection of Mandarin participants. Mandarin and English are supposed to differ in the vertical dimension of spatiotemporal metaphors. Surprisingly, those studies which comparatively investigated native English and Mandarin speakers’ temporal cognition all recruited ME bilinguals (e.g., college students studying in China or English-speaking countries) as Mandarin participants, while the researchers sought to examine the influence of native language on habitual thoughts. Given the research purpose and the potential effects of crosslinguistic distinctions on cognition, ME bilinguals could not constitute appropriate and representative samples of Mandarin speakers. Additionally, statistics reported by Chen (2019) indicate that at present literate Mandarin monolinguals account for approximately 30% of the Mandarin population, and over 80% of Mandarin monolinguals are adults above the age 40. Therefore, any studies which intend to involve a sample of Mandarin monolinguals had better exclude Chinese undergraduate/postgraduate students (around age 18–35), given that currently almost all Chinese college students are ME bilinguals.

While the first issue touches upon the relationship between L1/monolingualism and thought, the second question concerns the effect of L2/bilingualism on cognition. Across the existing investigations on ME bilingualism and temporal cognition (e.g., Boroditsky, 2001; Fuhrman et al., 2011; Miles et al., 2011; Lai and Boroditsky, 2013), researchers’ theoretical presupposition and the interpretation of data pertinent to ME bilinguals’ mental representations of time seem somewhat problematic. These studies shared a common logical assumption that Mandarin speakers have a strong propensity to think about time vertically between the dual sagittal vertical space–time mappings (Boroditsky, 2001; Chan and Bergen, 2005; Boroditsky et al., 2011; Fuhrman et al., 2011) and that the vertical axis may even be the only MTL for a Mandarin speaker (Grace and Vogel, 2008; Liu and Zhang, 2009). Therefore, Boroditsky (2001) and Fuhrman et al. (2011) maintained that Mandarin speakers’ vertical bias was found to be weakened as a consequence of learning L2 English which almost exclusively encodes time flow through sagittal spatiotemporal metaphors. Miles et al. (2011) two experiments demonstrated that ME bilinguals possessed both a vertical and a sagittal MTL, and the occurrence of an additional sagittal line, as argued by the authors, was manipulated by linguistic conventions of L2 English. In fact, all these claims are ill-grounded. According to the search results from CCL corpus (A corpus developed by the Center for Chinese Linguistics, the largest Mandarin Chinese corpus in the world), 80.35% of spatiotemporal metaphors in Mandarin are sagittal and 19.65% are vertical (Xiao, 2012). Therefore, sagittal spatiotemporal metaphors are used far more frequently than vertical metaphors in Mandarin. If a person’s native language serves as the primary source in shaping habitual thought, a Mandarin monolingual should have two MTLs, that is, one sagittal line and one vertical line, with the sagittal axis being the relatively dominant one (i.e., a sagittal bias in temporal reasoning). Therefore, it is expected that a Mandarin speaker has two MTLs (the sagittal timeline in particular) not because he/she has acquired L2 English, but because there are sagittal and vertical expressions of time in Mandarin. The coexistence of sagittal and vertical space–time mappings uncovered by Miles et al. (2011) is, in reality, indicative of no cognitive restructuring in ME bilinguals’ mental representations of time as compared with Mandarin monolinguals, which contradicted the authors’ original claims. Moreover, other researchers’ interpretations that Mandarin speakers’ vertical bias was attenuated as a result of acquiring L2 English (Boroditsky, 2001; Fuhrman et al., 2011) are untenable as well. In line with the linguistic patterns, Mandarin speakers should prioritize a sagittal space–time association over a vertical one. If Mandarin monolinguals do not have vertical bias at all, there would be no such phenomenon as the attenuation of vertical bias in ME bilinguals. Taken together, the preliminary experimental findings in hand cannot be counted as evidence for the view that acquisition of L2 English could restructure temporal cognition in ME bilinguals.

### Rationale for the Present Study

Supposing that the acquisition of L2 English can affect Mandarin speakers’ habitual thinking modes in the domain of time, the magnitude of the effects may be variant. According to Bassetti and Cook (2011) and Pavlenko (2011), acquiring and using an L2 with contrasting grammatical distinctions from the L1 may presumably generate profound consequences for non-linguistic cognition, but this kind of effect could be modulated by factors, such as age of onset of L2 acquisition (Harris, 2004; Ameel et al., 2005; Bylund and Jarvis, 2011), duration and type of residence in L2 speaking countries (Cook et al., 2006; Athanasopoulos, 2009), L2 proficiency/frequency of L2 use (Athanasopoulos and Kasai, 2008; Park and Ziegler, 2014), language contact (Bylund et al., 2013), context of acquisition (Smith and Samuelson, 2006; Kurinski and Sera, 2011), bilingual language mode (Kersten et al., 2010; Filipović, 2011), and the extent of dissimilarity/overlap between L1 and L2 (Scheutz and Eberhard, 2004; Roberson et al., 2005; Kousta et al., 2008). Pavlenko (2011) suggests that the bilingual mind can undergo different processes of cognitive restructuring depending on where the speaker falls on the continuum of monolingualism and bilingualism. As speakers learn and use an L2, novel perspectives and frames of reference from the L2 may be internalized, leading to the restructuring of the frames and categories of the speaker’s habitual modes of thinking developed by L1. This cognitive change can be manifested in the bilingual mind in several ways. Sometimes bilinguals perform more like monolinguals of L1 than monolinguals of L2. Sometimes bilinguals resemble monolinguals of L2 rather than monolinguals of L1. On most occasions, the conceptual representations of bilinguals do not fully correspond to either an L1-based or L2-based concept, exhibiting what is called “in-between performance” or “conceptual convergence” (Pavlenko, 2011, p. 247). In short, the relationship between bilingualism and cognition in general, and the process of cognitive restructuring in particular, could be dynamic and flexible in nature. What is clear thus far, however, is that bilinguals indeed to a greater or lesser degree think differently from monolinguals of L1 in most cognitive domains tested (Bassetti and Cook, 2011).

As elucidated above, English speakers talk about time almost exclusively *via* sagittal spatial terms, whereas Mandarin speakers rely on both sagittal and vertical spatiotemporal metaphors. A lot of previous studies have noticed the putative discrepancies between Mandarin and English in spatiotemporal metaphors, but the remarkable crosslinguistic similarities between the two languages have usually been overlooked. The crosslinguistic commonalities in sagittal expressions of temporal information complicate the question as to whether L2 English spatiotemporal metaphor is powerful enough to reshape ME bilinguals’ mental representations of temporal sequences, that is, whether Mandarin speakers’ sagittal conception of time is strengthened and vertical representation is attenuated as a result of acquiring L2 English. In addition, previous research (e.g., Boroditsky, 2001; Fuhrman et al., 2011) has already investigated the effects of extralinguistic or cultural variables, such as age of L2 acquisition, duration of stay in English-speaking countries on ME bilinguals’ temporal cognition, but they did not formally assess bilingual participants’ L2 proficiency which was found to constitute the most robust predictor of bilinguals’ cognitive shift in studies on other cognitive domains, such as color and space (Athanasopoulos and Kasai, 2008; Athanasopoulos et al., 2011; Kurinski and Sera, 2011; Park and Ziegler, 2014). Given that cognitive restructuring of bilinguals depends on the assumption that it may be properties of language *per se* rather than sociocultural variables that induce greater impact on the cognitive performance of bilingual speakers, it is important to rigorously assess L2 proficiency and to closely examine the effect of L2 proficiency on ME bilinguals’ mental representations of time.

We conducted two experiments to comparatively study mental representations of time along the sagittal and the vertical axis in English monolinguals, Mandarin monolinguals and ME bilinguals. Experiment 1 which recruited English and Mandarin monolinguals as participants aimed to confirm previous findings and establish that English and Mandarin speakers do think about time differently. Experiment 2 compared Mandarin monolinguals and ME bilinguals with different English proficiency levels, seeking to clarify whether the acquisition of L2 English could restructure Mandarin speakers’ temporal cognition and to expand the scope of existing literature by exploring if L2 proficiency *per se* can drive such a cognitive effect. In particular, we closely examined if cognitive restructuring (on condition that it indeed takes place) is susceptible to the influence of L2 proficiency, for example, if ME bilinguals at low English proficiency level pattern with Mandarin monolinguals and ME bilinguals at high English proficiency level accord with English monolinguals in mental representations of time. Note that the present study is unable to exhaust all aspects of temporal conceptualizations. Given the aims and scope of the present study, temporal cognition or mental representations of time is confined to mental representations of temporal sequences, that is, sequential time. Issues regarding temporal cognition in the present study are whether temporal sequences are spatially represented along a sagittal or vertical axis and what the directionalities are. More specifically, we ask if English and Mandarin speakers (and ME bilinguals as well) think about time along a sagittal or vertical axis and what the preferred orientations along each timeline are (i.e., front-to-back or back-to-front along the sagittal line, top-to-bottom or top-to-bottom along the vertical axis).

## Experiment 1

### Methods

#### Participants

We recruited 58 participants as potential candidates of Mandarin monolinguals from P. R. China and 44 participants as potential candidates of English monolinguals (25 females, *M*_age_ = 42.03, SD_age_ = 1.11) from the UK. Participants took part in Experiment 1 in exchange for payment or gifts. Prior to the experiment, all of them completed an L2 experience/proficiency questionnaire. In this questionnaire, participants were asked to answer if there are any languages other than their native language that they know. If the answer is yes, they had to list these non-native languages and to report their L2 proficiency level in these languages on a scale from 1 to 4 (1 = know very little about the language, 2 = elementary, 3 = intermediate, 4 = advanced). 54 Mandarin participants reported that they were Mandarin monolinguals, because they did not know any languages other than Mandarin. Four Mandarin participants could speak a little English, as they reported their L2 proficiency level to be 1 out of 4. Therefore, the four participants were excluded, resulting in a total of 54 Mandarin monolinguals as the sample (22 females, *M*_age_ = 44.81, SD_age_ = 1.67). As for the English participants, none of them reported knowing additional languages other than English. All participants reported to be right-handed and had normal or corrected-to-normal vision. They had already obtained a degree in tertiary education, thus having reached a high level of literacy in their native language.

#### Ethics Approval and Consent

All procedures were approved by the ethics committee of Yangzhou University and written informed consent was obtained from all participants.

#### Materials

**Figure 1:**
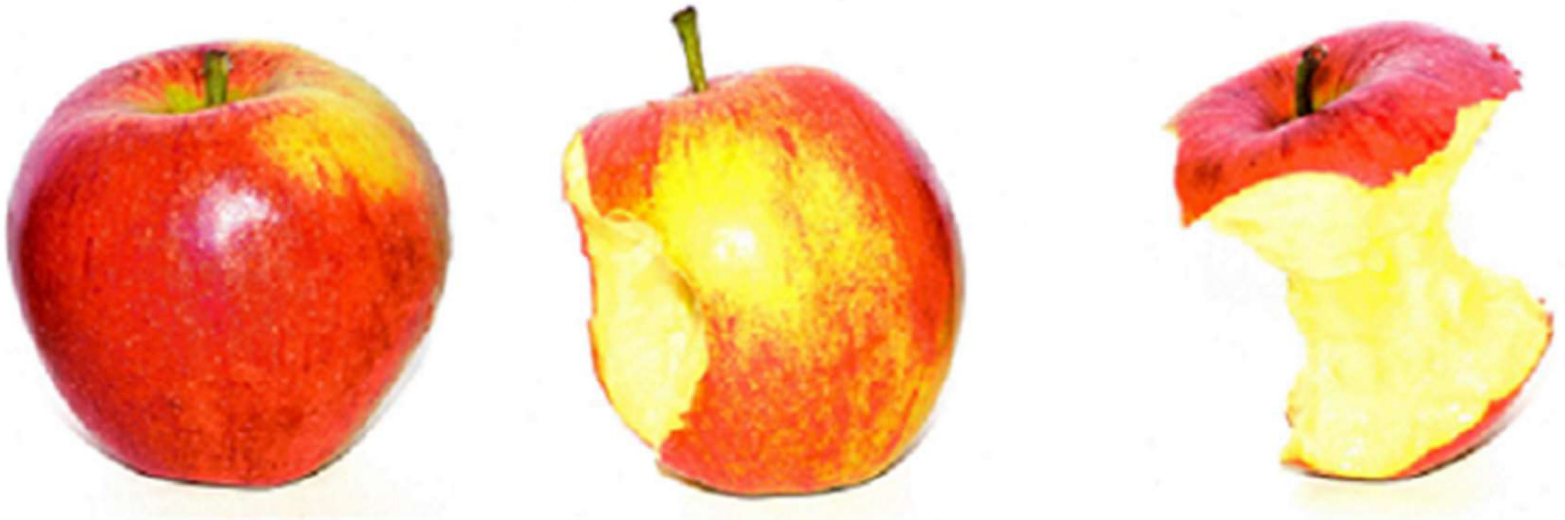
Examples of materials used in Experiment 1. The three images are (from left to right): a whole apple (the “early” time point), a half-eaten apple (the “middle” time point), and an apple core (the “late” time point).

Materials for the formal testing trials comprised 48 triplets of pictures, all describing themes of temporal progression. These triplets of pictures included 48 specific themes of temporal sequences (e.g., an apple being eaten, a film star aging, a pig growing). Within each theme, the triplet of pictorial stimulus showed a natural event at three different temporal stages. The three pictures represented an “early,” “middle,” and “late” time point, respectively. For example, in the “apple being eaten” theme, the “early” picture depicted “a whole apple,” the “middle” picture “a half-eaten apple,” and the “late” picture “an apple core” (please refer to Figure 1 as examples of the pictures). Since each temporal theme appeared twice (as is detailed below in the “Procedures” section) in the formal testing trials, a total of 96 stimuli was constructed. To design the materials, we consulted relevant studies (Boroditsky et al., 2011; Fuhrman et al., 2011) so that the themes of temporal progression depicted in our stimuli are similar to those in Boroditsky et al. (2011) and Fuhrman et al. (2011). But we did not adopt the materials directly from previous publications. All the stimuli in our study are original and are created by ourselves.

#### Procedures

All participants were tested individually in a quiet room, and all using the same desktop computer. On each trial, a red fixation cross was presented in the center of the screen for 600 ms. Then, the pictorial stimulus depicting the “middle” time point from one of the 48 critical sequences (e.g., a half-eaten apple) appeared in the center of the screen for 2,500 ms, followed by either a picture of the “early” time point (e.g., a whole apple) or the “late” time point (e.g., an apple core). Participants were instructed to decide whether the second picture presented showed an earlier or later time point than the first picture. The picture of the “early” or “late” time point would remain on the screen until participants made their judgments. Upon entry of a response, a blank screen of 100 ms replaced the stimulus and a new trial began. Instructions were presented in participants’ respective native language.

**Figure 2:**
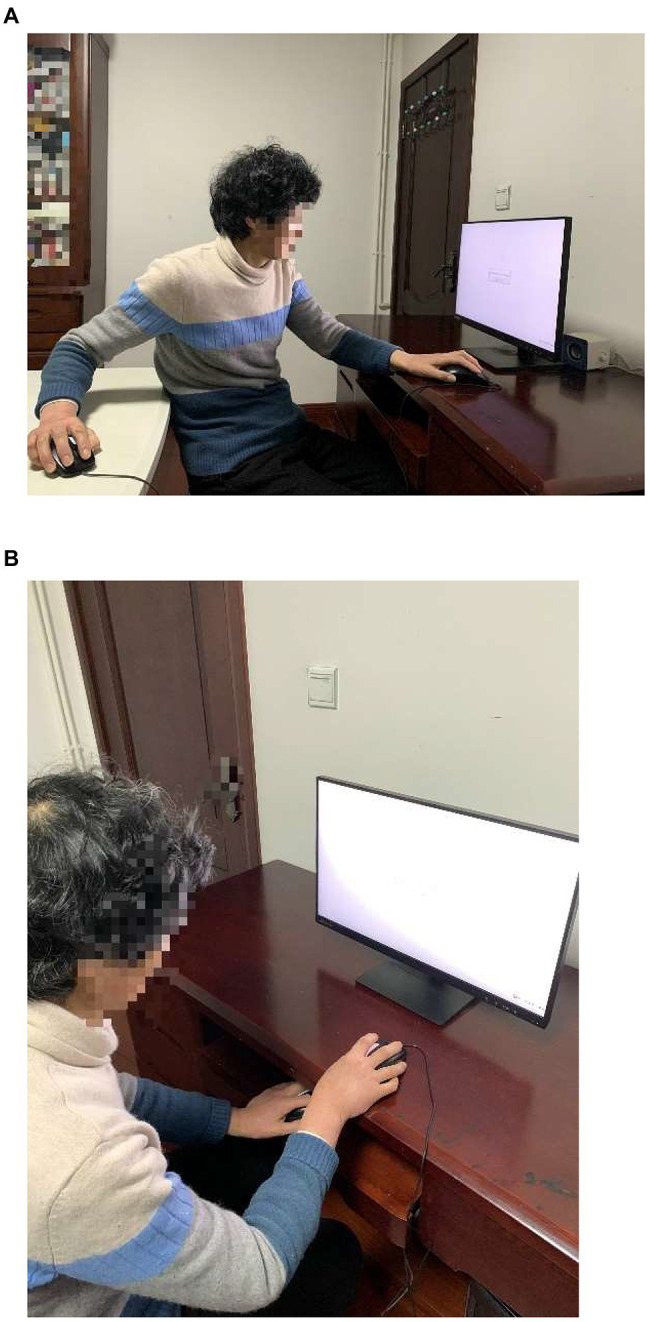
The position of the computer mice when making judgments along the sagittal axis **(A)** and along the vertical axis **(B)**.

Participants held two computer mice, one in each hand, and they needed to respond as quickly and accurately as possible by clicking one of the two mice. Different color stickers were placed on each mouse (pink and blue) and all response instructions referred only to the colors and not to the hand. To collect responses along the sagittal axis, participants held one mouse on the table in front of their body and the other mouse on the table behind their back (see Figure 2A). We counterbalanced which hand (right or left) was held in front across participants. For the vertical axis, we had participants hold one mouse above the table and the other mouse below the table (see Figure 2B). We also counterbalanced which hand (right or left) was held above across participants. Both the sagittal and the vertical arrangement of mice positions contained two conditions which were opposite to each other in designating the directionalities of time flow. In one of the sagittal condition, the pink mouse (i.e., the mouse in front) was designated as “earlier” and the blue mouse (i.e., the mouse on the back side) as “later” while in the other sagittal condition the mouse assignment was reversed (i.e., the pink mouse in front represented “later” and the blue mouse on the back represented “earlier”). In one of the vertical condition, the pink mouse (i.e., the mouse above) was designated as “earlier” and the blue mouse (i.e., the mouse below) as “later” while in the other vertical condition this mapping was reversed.

Each participant completed four mouse mapping blocks, each consisting of 24 trials: two blocks on the sagittal axis (one with the front mouse as “earlier” and the back mouse as “later” and one with the reverse mouse mapping) and two blocks on the vertical axis (top as “earlier” vs. down as “earlier”). For brevity, the four types of mouse mapping in the subsequent text will be referred to as abbreviations. The front-earlier/back-later mouse mapping is abbreviated as FE, back-earlier/front-later as BE, top-earlier/down-later as TE, and down-earlier/top-later as DE. The block order was counterbalanced across participants. Each of the “earlier” and “later” pictures of every temporal theme appeared once, respectively, in one of the two blocks along either of the two axes. For instance, if participants saw the “earlier” picture in the sagittal FE block, the “later” picture of the same sequence would appear in the BE block of the sagittal axis. The same temporal theme would not appear again on the vertical axis. The order of the trials was randomized within each block. Each block started with 6 additional practice trials, and the items used in the practice trials were not used subsequently in the formal testing trials. There is no limitation in the number of times for doing practice trials.

### Data Analysis and Results

Response accuracy was measured by the experimental program in following ways. When the second image on the screen depicted a time point earlier than the first image (i.e., a middle time point), participants had to click the mouse representing “earlier” which would result in a correct response. If participants clicked the mouse representing “later,” the response would be incorrect. Likewise, when the second image on the screen depicted a time point later than the first image, the correct answer required that participants clicked the “later” mouse. If participants clicked the earlier mouse, the response would be incorrect. Results from participants whose accuracy rate was lower than 90% (four English participants and three Mandarin participants) were excluded from the dataset. Trials which recorded a response latency farther than 3 SD away from each participant’s mean, respectively, on the four testing blocks (5.21%) and trials on which participants made errors (4.64%) were omitted from the RTs’ analyses. The accuracy rate was 94.83 and 95.9% for English monolinguals and Mandarin monolinguals, respectively.

The remaining response data were submitted to 2 × 2 × 2 mixed ANOVAs,^2^ with Spatial Axis (sagittal vs. vertical) and Directionality of Mouse Mapping (FE and TE vs. BE and DE) as the within-participants factors, and Language (English vs. Mandarin) as the between-participants factor. The results revealed a significant main effect of Language [*F*1 (1, 89) = 25.51, *p* < .001, $\eta_{p}^{2}$ = .209; *F*2 (1, 46) = 91.62, *p* < .001, $\eta_{p}^{2}$ = .666], of Spatial Axis [*F*1 (1, 89) = 7.29, *p* = .008, $\eta_{p}^{2}$= .076; *F*2 (1, 46) = 27.56, *p* < .001, $\eta_{p}^{2}$ = .375], and of Directionality of Mouse Mapping [*F*1 (1, 89) = 81.25, *p* < .001, $\eta_{p}^{2}$= .477; *F*2 (1, 46) = 87.90, *p* < .001, $\eta_{p}^{2}$ = .656]. A significant three-way (Language × Spatial Axis × Directionality of Mouse Mapping) interaction was also observed [*F*1 (1, 89) = 7.83, *p* = .006, $\eta_{p}^{2}$ = .081; *F*2 (1, 46) = 4.92, *p* = .032, $\eta_{p}^{2}$ = .097].

To examine the results in more detail, we conducted 2 × 2 mixed ANOVAs (2 language × 2 directionality of mouse mapping) for the sagittal axis and the vertical axis separately.

#### The Sagittal Axis

2 × 2 mixed ANOVAs, with Language as the between-participants factor, and Directionality of Mouse Mapping (FE vs. BE) as the within-participants factor, revealed a significant main effect of Language [*F*1 (1, 89) = 8.04, *p* = .006, $\eta_{p}^{2}$ = .083; *F*2 (1, 46) = 81.42, *p* < .001, $\eta_{p}^{2}$ = .639], and of Directionality of Mouse Mapping [*F*1 (1, 89) = 81.53, *p* < .001, $\eta_{p}^{2}$ = .478; *F*2 (1, 46) = 116.56, *p* < .001, $\eta_{p}^{2}$ = .717]. A non-significant Language × Directionality of Mouse Mapping interaction, *F*1 (1, 89) = 1.05, *p* = .307; *F*2 (1, 46) = .36, *p* = .549, was observed. Planned paired *t*-tests showed that both English and Mandarin monolinguals responded to FE mouse mapping condition significantly faster than to BE condition along the sagittal axis [English monolinguals: *t*1 (39) *=* −4.97, *p* < .001, *d* = .784; *t*2 (23) *=* −6.29, *p* < .001, *d* = 1.26, and Mandarin monolinguals: *t*1 (50) *=* −10.17, *p* < .001, *d* = 1.42; *t*2 (23) *=* −12.02, *p* < .001, *d* = 2.45].

#### The Vertical Axis

**Figure 3:**
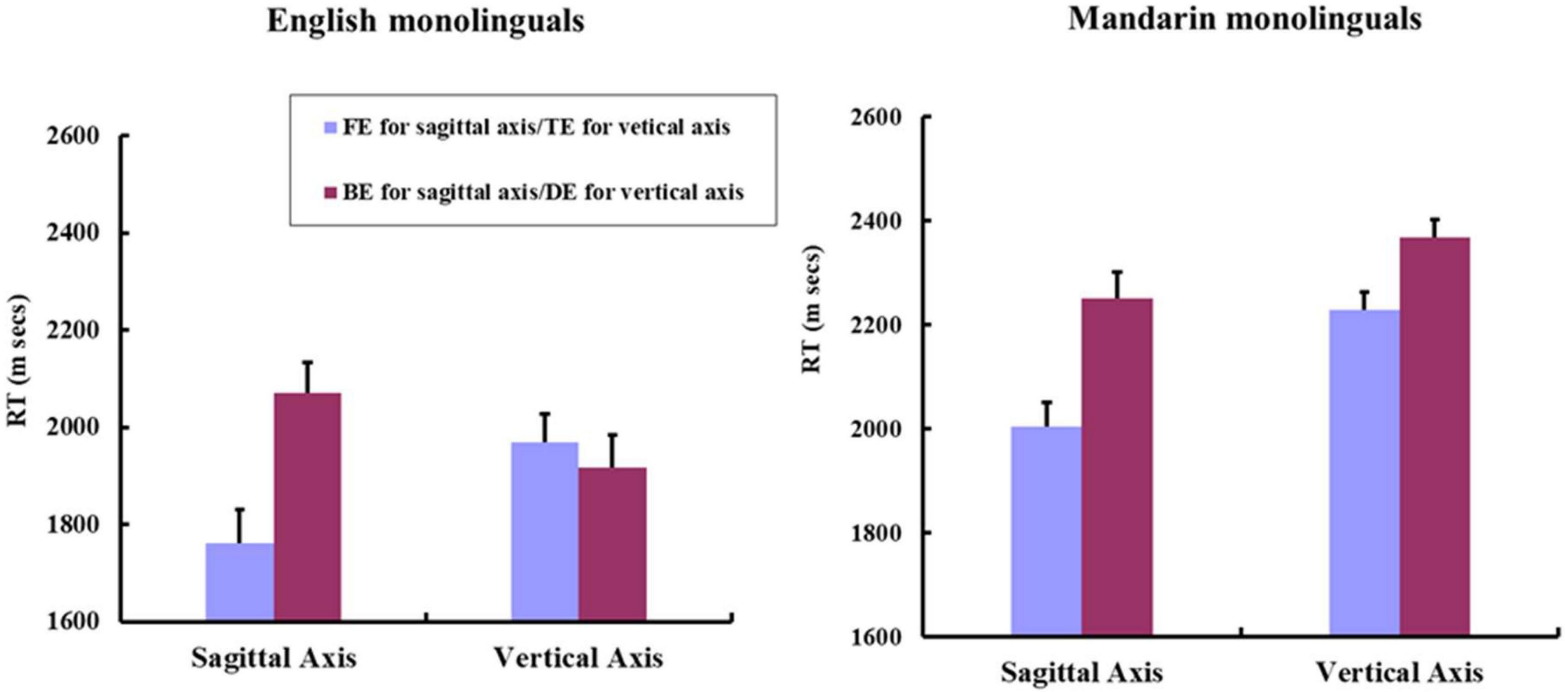
Mean RTs for FE and BE mouse mappings along the sagittal axis; TE and DE mouse mappings along the vertical axis by English and Mandarin monolinguals. The figure plotted by participants’ mean RTs. Error bars indicate standard errors of the mean.

2 × 2 mixed ANOVAs, with Language as the between-participants factor, and Directionality of Mouse Mapping (TE vs. DE) as the within-participants factor, revealed a significant main effect of Language [*F*1 (1, 89) = 31.94, *p* < .001, $\eta_{p}^{2}$ = .264; *F*2 (1, 46) = 69.82, *p* < .001, $\eta_{p}^{2}$ = .603]. A significant Language × Directionality of Mouse Mapping interaction was observed [*F*1 (1, 89) = 12.58, *p* = .0062, $\eta_{p}^{2}$ = .124; *F*2 (1, 46) = 13.61, *p* = .0059, $\eta_{p}^{2}$ = .228]. Planned paired *t*-tests showed that Mandarin monolinguals responded significantly faster to TE than to DE condition [*t*1 (50) *=* −5.76, *p* < .001, *d* = .806; *t*2 (23) *=* −3.41, *p* = .0024, *d* = .696], while English monolinguals showed no such difference between the two conditions [*t*1 (39) *=* .98, *p* = .33; *t*2 (23) *=*1.48, *p* = .153]. Figure 3 plotted the mean RTs for the four different mouse mapping conditions along the sagittal and the vertical axes by English and Mandarin monolinguals.

English and Mandarin monolinguals exhibited different patterns in the task. Both English and Mandarin monolinguals responded faster when the “earlier” mouse was in front than when it was on the back in the sagittal response condition. In the vertical condition, only Mandarin speakers responded faster when the “earlier” mouse was on the top than when it was on the bottom, and there was no such difference for English monolinguals. As shown in Figure 3, results demonstrated that the magnitude of MTL effect (i.e., RTs in the FE condition minus RTs in the BE condition along the sagittal axis; RTs in the TE condition minus RTs in the DE condition along the vertical axis) was larger in the sagittal axis (−246 ms) than in the vertical axis (−141 ms). It is also noteworthy that the coexistence of two MTLs may compete with each other for cognitive resources in Mandarin monolinguals’ conceptual system. Such kind of competition may cause interference in processing spatiotemporal associations along different spatial axes in the task, and Mandarin monolinguals’ response would accordingly slow down. This possibility might explain the overall RTs differences between Mandarin and English participants in Experiment 1 (as can be seen from Figure 3), given that RTs by Mandarin monolinguals were, on the whole, shorter than those by English monolinguals.

There is an important conceptual issue that requires clarification, that is, the concept of time examined in our two experiments is sequential time rather than deictic time. Time is not a unitary concept, but a myriad of different processes (e.g., representations of temporal duration, temporal sequence, and temporal orientation). Insofar as different types of relationship between temporal events are concerned, time can be classified into deictic time and sequential time (Núñez and Cooperrider, 2013). Deictic time is employed when speakers locate a temporal period with respect to the deictic center (the present moment “now,” also called the “ego”), reflecting past/future relationships (e.g., *back* in those days, bright future *ahead* of us). Sequential time, on the other hand, establishes a posteriority/anteriority relation between two or more temporal events that are referenced relative to each other, indicative of earlier/later relationships (e.g., the calm *before* the storm, Thursday comes *after* Wednesday). In terms of the experimental materials and design, the concept of time under investigation in the present study accords with sequential time, as the triplet of pictorial stimulus depicted a natural event at three different temporal stages (the three pictures represented “early,” “middle,” and “late” time points, respectively) and the responses call for “earlier/later” decisions. The distinction between deictic and sequential time brings about bidirectional constructions of sagittal time axis in languages. Deictic time proceeds from back to front, with past on the back and future on the front side (e.g., way *back* in 1960s, *ahead* in the future). However, the orientation is directed at an opposite pattern for sequential time, because it flows from front to back (Tenbrink, 2011; Bender and Beller, 2014), with earlier event on the front and later event on the back side (e.g., Tuesday comes *before* Wednesday, Friday comes *after* Thursday). The predominant recruitment of front/earlier and back/later for sequential time along the sagittal axis can be found in both corpus studies on English and Mandarin (Xiao, 2012; Walker et al., 2017) and behavior experiments (Walker et al., 2014, 2017; Yang et al., 2020). These observations may explain why RTs were shorter in FE than in BE condition along the sagittal axis in the present study.

English monolinguals rely on a sagittal front-to-back line to think about time, while Mandarin monolinguals access both a front-to-back and a vertical top-to-bottom representation of temporal information. Moreover, the greater magnitude of MTL effect in the sagittal axis than in the vertical axis indicates that the sagittal axis occupies a relatively dominant role between the two MTLs in Mandarin monolinguals. The specific space–time mappings in English and Mandarin monolinguals’ minds were congruent with linguistic patterns of spatiotemporal metaphors for sequential time in English and Mandarin, respectively. Overall, results of Experiment 1 confirm previous findings that English and Mandarin speakers do think about time differently. Notably the general conclusion yielded from foregoing studies that English and Mandarin speakers think about time differently has to be specified. English and Mandarin speakers resemble each other in their representations of a sagittal front-to-back timeline, but Mandarin speakers simultaneously accommodate a vertical top-to-bottom MTL in their minds.

## Experiment 2

Since Experiment 1 showed that English and Mandarin monolinguals think about time differently (especially in the vertical representations of time flow), we further performed Experiment 2 to test whether the acquisition of L2 English could reshape Mandarin speakers’ mental representations of temporal sequence. In light of the crosslinguistic differences/similarities between the two languages in spatiotemporal metaphors and the potential effect of L2 proficiency on habitual thought, we envisage two possibilities concerning the strength of L2 English that may contribute to the reconstruction of ME bilinguals’ temporal cognition. First, L2 acquisition does not affect habitual thought (i.e., thinking patterns exhibited by monolingual speakers of L1) at all. In this case, the influence of L2 English would not be extended to Mandarin speakers’ conceptual system. ME bilinguals, regardless of their English proficiency levels, would accord with Mandarin monolinguals in their mental representations of time. Second, the magnitude of L2 influence is variable, depending on L2 proficiency. For ME bilinguals of low English proficiency, L2 exerts moderate influence on habitual thought. On this occasion, ME bilinguals would slightly reinforce the sagittal space–time mapping while preserving the vertical MTL. In other words, their conceptions of time pattern with more of Mandarin monolinguals’ than English monolinguals’. For ME bilinguals of advanced L2 or even native-like L2 English proficiency, habitual modes of thought are subject to profound L2 influence or systematically restructured by L2 linguistic forces. On this occasion, L2 would pervade cognition so that the sagittal MTL may occupy a dominant role and that the vertical MTL may become inactive or even be erased in consequence of the cognitive restructuring. Therefore, ME bilinguals would align with more of English monolinguals than Mandarin monolinguals in their temporal thinking patterns. The purpose of Experiment 2 was to test which of the two hypotheses could be supported by experimental evidence.

### Methods

#### Participants

Forty-eight potential candidates of Mandarin monolinguals were pre-registered for Experiment 2. As shown in the self-report of an L2 experience/proficiency questionnaire (the same as the questionnaire used in Experiment 1), six candidates who reported to know an L2 (i.e., English), with very limited level of proficiency (1 out of 4 in the rating scale), were excluded from the sample prior to Experiment 2. None of the remaining 42 participants reported to know any additional language other than Mandarin, and they were chosen as Mandarin monolinguals (19 females, *M*_age_ = 44.21, SD_age_ = .77). As for the selection of ME bilinguals, we recruited 112 participants as potential candidates of ME bilinguals with low English proficiency, and 156 participants as potential candidates of ME bilinguals with high English proficiency. These participants reported to only know English as an L2. We also asked them to take a mock IELTS test^3^ so that we could screen these candidates and select the most appropriate participants who were indeed bilinguals with low/advanced L2 proficiency. Those who gained an IELTS score ranging from 3 to 4 were finally chosen as ME bilinguals with low English proficiency (*N* = 39, 11 females, *M*_age_ = 45.97, SD_age_ = 1.69). Those who gained an IELTS score 8 or above were finally chosen as ME bilinguals with high English proficiency (*N* = 34, 18 females, *M*_age_ = 43.06, SD_age_ = 1.28). All participants came from P. R. China. They reported to be right-handed and had normal or corrected-to-normal vision. They received payments as rewards for their participation. All of them had already obtained a degree in tertiary education, thus having reached a high level of literacy in Mandarin. To calculate the sample size, we assumed a medium-sized effect ($\eta_{p}^{2}$ = .06) of the interaction between English proficiency group and mouse mapping type, alpha level of .05, along with default sample correlation and non-sphericity values (Faul et al., 2007), which yielded a recommended sample size of 33 participants. We thus slightly oversampled, factoring in certain attrition.

#### Ethics Approval and Consent

All procedures were approved by the ethics committee of Yangzhou University and written informed consent was obtained from all participants.

#### Materials and Procedures

All materials and procedures were identical to Experiment 1, with the exception of the language of instructions. Instructions were presented in Mandarin for Mandarin monolinguals, but in L2 English instead of L1 Mandarin for ME bilinguals. Some previous publications suggested that bilinguals can switch between different representations of time depending on contextual cues (e.g., Fuhrman et al., 2011; Chen and O’Seaghdha, 2013; Bylund and Athanasopoulos, 2017). As revealed by the performance in the experimental tasks, bilinguals were more likely to approach monolinguals of L2 in temporal cognition if they were instructed in L2 rather than L1 (Fuhrman et al., 2011; Chen and O’Seaghdha, 2013). Given these observations, the experimenter in the present study provided ME bilinguals with instructions in L2 English to maximize the possibility that ME bilingual participants behave like native speakers of English (i.e., ME bilinguals’ performance show cognitive restructuring in the experiment). In addition, the experimenter demonstrated the procedures of the task by PowerPoint slides before participant formally entered the experimental program, which guaranteed that every participant could understand how to complete the task.

#### Data Analysis and Results

Results from participants whose accuracy rate was lower than 90% (three Mandarin monolinguals, six ME bilinguals with low English proficiency and thirteen ME bilinguals) with high English proficiency were excluded from the dataset. Trials which recorded a response latency farther than 3 SD away from each participant’s mean, respectively, on the four testing blocks (7.62%) and trials on which participants made errors (5.82%) were omitted from the RTs’ analyses. The accuracy rate was 95.22, 93.43, and 93.89% for Mandarin monolinguals, ME bilinguals with low English proficiency and ME bilinguals with high English proficiency, respectively.

The remaining response data were submitted to 3 × 2 × 2 mixed ANOVAs, with Spatial Axis (sagittal vs. vertical) and Directionality of Mouse Mapping (FE and TE vs. BE and DE) as the within-participants factors, and English Proficiency (Mandarin monolinguals vs. bilinguals with low English proficiency vs. bilinguals with high English proficiency) as the between-participants factor. The results revealed a significant main effect of Proficiency [*F*1 (2, 92) = 11.69, *p* < .001, $\eta_{p}^{2}$ = .203; *F*2 (2, 69) = 12.10, *p* < .001, $\eta_{p}^{2}$ = .260], of Spatial Axis [*F*1 (1, 92) = 20.72, *p* < .001, $\eta_{p}^{2}$ = .184; *F*2 (1, 69) = 20.67, *p* < .001, $\eta_{p}^{2}$ = .230], and of Directionality of Mouse Mapping [*F*1 (1, 92) = 67.54, *p* < .001, $\eta_{p}^{2}$ = .423; *F*2 (1, 69) = 38.60, *p* < .001, $\eta_{p}^{2}$ = .359]. As can be seen from the results, the response latency for sagittal and vertical axes vary across Mandarin monolinguals, ME bilinguals with low English proficiency and ME bilinguals with high English proficiency. But all the three groups responded to FE condition faster than BE condition along the sagittal axis and to TE condition faster than DE condition. English proficiency, therefore, did not play a role that prompted participants to generate different response patterns in directionality of mouse mapping along each of the two spatial axes. This kind of consistency was confirmed in a non-significant three-way (English Proficiency × Spatial Axis × Directionality of Mouse Mapping) interaction (both *F*s < 1), which demonstrates that participants, regardless of their L2 proficiency levels, resembled one another when responding to FE/BE conditions along the sagittal axis and TE/DE conditions along the vertical axis.

To examine the results in more detail, we conducted 3 × 2 mixed ANOVAs (3 English Proficiency × 2 Directionality of Mouse Mapping) for the sagittal axis and the vertical axis separately.

##### The Sagittal Axis

3 × 2 mixed ANOVAs, with English Proficiency as the between-participants factor, and Directionality of Mouse Mapping (FE vs. BE) as the within-participants factor, revealed a significant main effect of Proficiency [*F*1 (2, 92) = 13.51, *p* < .001, $\eta_{p}^{2}$ = .227; *F*2 (2, 69) = 11.61, *p* < .001, $\eta_{p}^{2}$ = .252], and of Directionality of Mouse Mapping [*F*1 (1, 92) = 50.33, *p* < .001, $\eta_{p}^{2}$ = .354; *F*2 (1, 69) = 38.78, *p* < .001, $\eta_{p}^{2}$ = .360]. A non-significant English Proficiency × Directionality of Mouse Mapping interaction [both *F*s < 1] was observed. Planned paired *t*-tests showed that all three groups responded to FE mouse mapping condition significantly faster than to BE condition along the sagittal axis. Mandarin monolinguals [*t*1 (38) *=* −5.87, *p* < .001, *d* = .939; *t*2 (23) *=* −3.79, *p* = .0094, *d* = .776], ME bilinguals with low English proficiency [*t*1 (34) *=* −3.50, *p* = .0013, *d* = .594; *t*2 (23) *=* −3.02, *p* = .0061, *d* = .617], ME bilinguals with high English proficiency [*t*1 (20) *=* −3.67, *p* = .0015, *d* = .801; *t*2 (23) *=* −4.16, *p* < .001, *d* = .848].

##### The Vertical Axis

**Figure 4:**
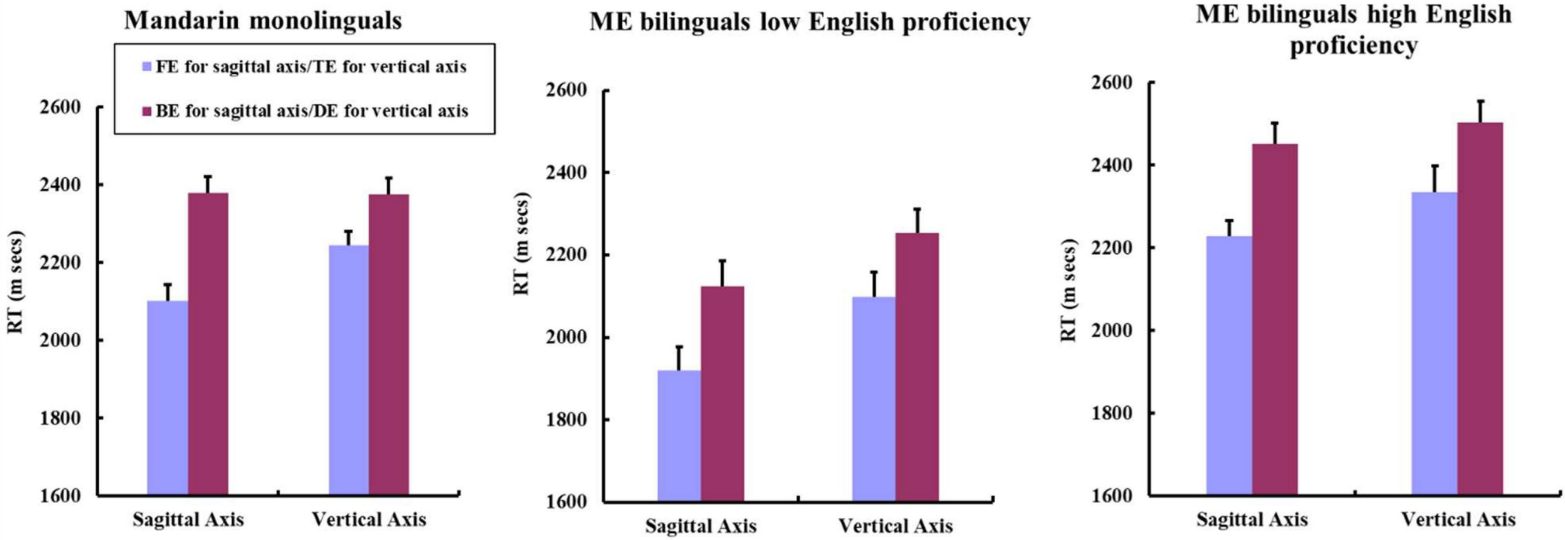
Mean RTs for FE and BE mouse mappings along the sagittal axis; TE and DE mouse mappings along the vertical axis by Mandarin monolinguals, ME bilinguals with low English proficiency and ME bilinguals with high English proficiency. The figure plotted by participants’ mean RTs. Error bars indicate standard errors of the mean.

3 × 2 mixed ANOVAs, with English Proficiency as the between-participants factor, and Directionality of Mouse Mapping (TE vs. DE) as the within-participants factor, revealed a significant main effect of Proficiency [*F*1 (2, 92) = 6.56, *p* < .01, $\eta_{p}^{2}$ = .125; *F*2 (2, 69) = 6.67, *p* < .01, $\eta_{p}^{2}$ = .162], and of Directionality of Mouse Mapping [*F*1 (1, 92) = 21.83, *p* < .001, $\eta_{p}^{2}$ = .192; *F*2 (1, 69) = 17.13, *p* < .001, $\eta_{p}^{2}$ = .199]. A non-significant English Proficiency × Directionality of Mouse Mapping interaction (both *F*s < 1) was observed. Planned paired *t*-tests showed that all three groups responded to TE mouse mapping condition significantly faster than to DE condition along the vertical axis. Mandarin monolinguals [*t*1 (38) *=* −2.62, *p* = .013, *d* = .42; *t*2 (23) *=* −2.21, *p* = .034, *d* = .452], ME bilinguals with low English proficiency [*t*1 (34) *=* −3.10, *p* = .0038, *d* = .524; *t*2 (23) *=* −2.57, *p* = .017, *d* = .525], ME bilinguals with high English proficiency [*t*1 (20) *=* −2.54, *p* = .020, *d* = .554; *t*2 (23) *=* −2.56, *p* = .017, *d* = .522]. Figure 4 plotted the mean RTs for the four different mouse mapping conditions along the sagittal and the vertical axes by Mandarin monolinguals and ME bilinguals at low/high English proficiency levels.

As can be seen from Figure 4, results also demonstrated that the magnitude of MTL effect was larger in the sagittal axis than in the vertical axis for Mandarin monolinguals (sagittal vs. vertical: −277 ms vs. −132 ms), ME bilinguals with low English proficiency (sagittal vs. vertical: −206 ms vs. −155 ms), and ME bilinguals with advanced English proficiency (sagittal vs. vertical: −224 ms vs. −168 ms).

Mandarin monolinguals, ME bilinguals with low English proficiency and ME bilinguals with high English proficiency displayed similar response pattern in the task. All of the three groups responded to FE condition faster than BE condition along the sagittal axis, and to TE condition faster than DE condition along the vertical axis. These results suggest that two MTLs coexist independently in each participant’s minds, one proceeding from front to back and one from top to bottom. In addition, the sagittal line occupies a relatively dominant role between the two MTLs, as the magnitude of MTL effect was greater in the sagittal axis than in the vertical axis for all three groups of Mandarin speakers.

Note that no cogent evidence yielded from Experiment 2 can substantiate the possibility that ME bilinguals gradually approach English monolinguals (i.e., exclusively rely on a sagittal line to represent time) or diverge from Mandarin monolinguals in their temporal thinking patterns with increasing L2 English proficiency. The magnitude of sagittal MTL effect was largest for Mandarin monolinguals, followed by ME bilinguals with high English proficiency and lastly by ME bilinguals with low English proficiency (this relative order regarding the magnitude of sagittal MTL effect for the three groups is abbreviated as: Mandarin monolingual > ME bilingual with high English proficiency > ME bilingual with low English proficiency). Meanwhile, comparing the magnitude of vertical MTL effect among the three groups revealed such a relative order: ME bilingual with high English proficiency > ME bilingual with low English proficiency > Mandarin monolingual. If cognitive restructuring indeed takes place and is subject to the influence of different L2 proficiency levels, the expected orders concerning the relative magnitude of MTL effects among the three groups should be: ME bilingual with high English proficiency > ME bilingual with low English proficiency > Mandarin monolingual on the sagittal axis; Mandarin monolingual > ME bilingual with low English proficiency > ME bilingual with high English proficiency on the vertical axis. However, current findings were inconsistent with these predictions. Mandarin speakers neither strengthened the sagittal space–time mapping nor inactivated the vertical spatiotemporal associations as a consequence of acquiring English and achieving higher-level proficiency in English. Taken together, results of Experiment 2 verified our first assumption concerning the strength of L2 English that may contribute to the reconstruction of ME bilinguals’ cognition, that is, the acquisition of L2 English does not affect Mandarin speakers’ mental representations of temporal sequence. ME bilinguals, regardless of their English proficiency levels, coincide with Mandarin monolinguals in their patterns of temporal cognition.

It should also be noted that the overall RTs for ME bilinguals of low English proficiency appeared to be shorter than the other two Mandarin groups. It is likely that this overall disparities in performance is simply due to differences in familiarity with the experiment task (for similar findings from relevant studies, see Boroditsky et al., 2011; Fuhrman et al., 2011). Our data record provided by E-Prime showed that 88% (29/33) of ME bilinguals with low English proficiency completed the practice trials twice or more, while only 21% (8/39) of Mandarin monolinguals and 19% (5/21) of ME bilinguals with advanced English proficiency did the practice trials more than once. More practices with the practice trials by ME bilinguals with low English proficiency may presumably bring about higher degree of familiarities with the task as compared with the other two Mandarin groups, which eventually results in overall shorter response latency for the formal testing trials. However, such differences in overall RTs (and perhaps familiarity with the task) did not undermine the results and conclusion of the present study. In analyzing the data yielded from the current experimental design, the critical comparison lies in the RTs difference between FE and BE mouse mapping condition along the sagittal axis and between TE and DE condition along the vertical axis, within each Mandarin group. Evidently, all three Mandarin groups exhibited similar response pattern, as each group responded to FE condition significantly faster than BE condition and to TE condition significantly faster than DE condition.

## General Discussion

The present study recruited English monolinguals, Mandarin monolinguals and ME bilinguals as participants to examine whether English and Mandarin speakers think about time differently and whether the acquisition of L2 English could reshape Mandarin speakers’ temporal cognition. We employed the temporal congruency categorization task in which participants saw pictures that appeared one after another on the computer screen. The first picture depicted events representing a middle time point (e.g., a famous film star in his/her mature years), followed by an early (a famous film star in his/her youth) or late time point (a famous film star in his/her old age). Participants had to judge if the second pictorial stimulus stood for an earlier or later time point than the first image by clicking one of two computer mice in his/her hand. In one of the sagittal condition, the front mouse was designated as “earlier” and the back mouse “later” while in the other sagittal condition the mouse assignment was reversed. Likewise, in one of the vertical condition the top mouse was designated as “earlier” and the bottom mouse “later” while in the other vertical condition this mapping was reversed. Across two experiments, Mandarin speakers were faster to make a decision in the FE condition than in the BE condition along the sagittal axis, and they also responded faster to TE condition than DE condition along the vertical axis. However, English speakers showed significant RTs difference between the two conditions along the sagittal axis only.

Results add to previous studies (Boroditsky, 2001; Chan and Bergen, 2005; Boroditsky et al., 2011; Fuhrman et al., 2011; Yang and Sun, 2016b) by corroborating that English and Mandarin speakers do think about time differently. While English monolinguals encoded passage of time into a sagittal front-to-back linear path, Mandarin monolinguals relied on both a sagittal and a vertical top-to-bottom spatial axis to reason about time. These space–time mappings in cognition can be approximately predicted by patterns in English and Mandarin spatiotemporal metaphors of sequential time, respectively. Therefore, current findings support the linguistic relativity hypothesis which suggests that one’s native language plays a privileged role in shaping habitual thought.

Nevertheless, such a Whorfian effect regarding the relationship between L2 and cognition was not identified in ME bilinguals. The L2 linguistic force *per se* did not appear to modulate Mandarin speakers’ temporal cognition. ME bilinguals, irrespective of their English proficiency levels, exhibited temporal thinking patterns matching those of Mandarin monolinguals. They neither approached English monolinguals nor deviated from Mandarin monolinguals, let alone showing the “in-between performance” that was simultaneously similar to and distinct from both L1 and L2 monolingual norms. These results of Experiment 2 revise the position with reference to ME bilinguals’ temporal reasoning proposed in foregoing studies (Boroditsky, 2001; Fuhrman et al., 2011; Miles et al., 2011), demonstrating that the acquisition of L2 English does not reshape Mandarin speakers’ mental representations of temporal sequence.

L2 proficiency did not appear to exert influence on ME bilinguals’ temporal cognition. But results of Experiment 2 give rise to another interesting question: Could factors, such as age of onset of L2 acquisition, frequency of L2 use, and duration of residence in L2 speaking countries, independently lead to cognitive restructuring of time in ME bilinguals? We assume that it is also unlikely for these elements to independently drive such an effect of reconstruction. For one thing, the ultimate consequence brought about by earlier age of L2 acquisition, more frequent L2 use and longer duration of staying in L2 speaking countries is approaching advanced or native-like L2 proficiency. For another, previous studies (Athanasopoulos and Kasai, 2008; Athanasopoulos et al., 2011; Kurinski and Sera, 2011; Park and Ziegler, 2014) manifested that it is ultimately linguistic competence *per se* (e.g., L2 proficiency) rather than other extralinguistic or sociocultural variables that is most significantly and directly associated with bilinguals’ cognitive shift. Since advanced/native-like L2 proficiency, the ultimate attainment and most powerful element, is unable to alter bilinguals’ conceptual representations in the present study, it would be much more difficult for those less potent factors to produce such an effect.

Although cognitive restructuring was not found in the domain of time for ME bilinguals, bilinguals of other languages were documented to undergo genuine cognitive reorganization or restructuring in domains, such as color (Athanasopoulos, 2009; Athanasopoulos et al., 2011), emotion (Harris, 2004; Besemeres, 2011), motion (Kersten et al., 2010; Czechowska and Ewert, 2011; Flecken et al., 2014, 2015; Gerwien and von Stutterheim, 2018), object categorization (Athanasopoulos and Kasai, 2008), and space (Park and Ziegler, 2014). For instance, Athanasopoulos and Kasai (2008) addressed whether English–Japanese bilinguals match objects based on color or shape, and results explicitly show that advanced bilinguals performed more like monolingual speakers of their L2, while intermediate bilinguals patterned more with speakers of their L1. Another example is a recent study (Park and Ziegler, 2014) which focused on whether adult Korean–English bilinguals categorize spatial concepts concerning containment, support, and contact differently from Korean and English monolinguals. Results demonstrate that bilingual participants resembled neither Korean nor English monolinguals, which is indicative of an ongoing conceptual restructuring that is taking place in the bilingual mind.

Why is there a striking discrepancy between the findings reported here on time and those on color, space, and motion, etc.? One possibility is that there are salient linguistic similarities between English and Mandarin in expressing temporal relations. Previous studies revealed that resemblance/distinction between two languages is one of the crucial ingredients that can affect the degree to which bilinguals undergo cognitive restructuring (Scheutz and Eberhard, 2004; Roberson et al., 2005). If the two languages contain analogous lexical and grammatical categories, bilinguals would exhibit performance akin to monolingual speakers of both L1 and L2. As for the comparison between Mandarin (a Sino-Tibetan language) and English (an Indo-European language), the two languages indeed comprise contrasting lexical and grammatical categories. However, Mandarin and English also resemble each other in at least one respect, that is, the frequent use of sagittal spatiotemporal metaphors. Because Mandarin speakers themselves do explicitly access sagittal space–time mapping without acquiring L2 English, it is unnecessary for ME bilinguals to adjust to L2 norms by substantially reinforcing the sagittal timeline and inactivating the vertical line in the mind. The crosslinguistic commonalities minimize the possibility that Mandarin speakers experience cognitive change as a result of English acquisition, even if there are significant crosslinguistic disparities in the vertical dimension of expressing time. Given the coexistence of pronounced similarities and distinctions between English and Mandarin in spatiotemporal metaphors, the relationship between ME bilingualism and cognition in the domain of time should be, in principle, a complicated linguistic and psychological issue. We assume that this kind of convergence between L1 and L2 temporal thinking patterns can also be found in bilingual speakers of other Indo-European languages (e.g., Dutch–English, German–English, Italian–English, and Spanish–English bilinguals). In the past years a large body of research examined how native speakers of Dutch, German, Italian, Spanish think about time (e.g., Ouellet et al., 2010; Ulrich et al., 2012; Casasanto and Bottini, 2014; Vallesi et al., 2014; Callizo-Romero et al., 2020), and participants were shown to conceive of time as a sagittal-oriented spatial axis. It is noteworthy that a number of participants in these studies were bilinguals of L2 English (e.g., German–English bilinguals, Spanish–English bilinguals). Their temporal thinking patterns are expected to correspond to those possessed by both monolinguals of L1 and L2. This assumed congruency may simply arise from the lexical similarities between English and other Indo-European languages under investigation, as all these languages predominately depict time as flowing along a sagittal plane. In light of the linguistic overlaps, we envisage that native speakers of Dutch, Spanish, German and Italian do not need to reconstruct their temporal cognition in the process of acquiring L2 English. This hypothesis can be submitted to experimental tests by further studies.

A second possibility is that time is a more abstract concept in comparison with color, space and motion, and many properties of time (e.g., sagittal or vertical directionality in which time flows) are unextractable from our sensory experience with the physical world (Boroditsky, 2001). Perhaps these aspects can only be encoded in one’s native language—most often through spatiotemporal metaphors. Given this constraint, a person has to predominately count on his/her native language when acquiring the concept of time. In other words, mental representations of time have become intimately intertwined with a person’s native language early since his/her childhood. Consequently, temporal cognition may be fixed once-and-for-all by L1 and cannot be easily transformed by the acquisition of an additional language. On the contrary, concepts, such as color, motion, and space, are more concrete and reliant on sensory experience, demonstrating a much higher degree of flexibility and dynamicity. Because of their plasticity in nature, cognitive domains including color, motion, and space can be more susceptible to the influence of L2 forces.

Given that Experiment 2 focused on whether the acquisition of L2 English could reshape Mandarin speakers’ mental representations of time, further studies may consider examining the effect of L2 Mandarin on L1 English in temporal cognition for bilinguals with L1 English and L2 Mandarin (i.e., English–Mandarin bilinguals). Findings of the present study indicate that native Mandarin speakers did not adjust to L2 English norms by inactivating or erasing the vertical MTL after they acquired L2 English. However, the procedure of L2 Mandarin acquisition for native English speakers may not be entirely the same as that of L2 English acquisition for native Mandarin speakers. It remains an open question as to whether the acquisition of L2 Mandarin could reconstruct native English speakers’ temporal cognition and add an additional vertical timeline in their minds. This interesting issue is worth exploration by follow-up studies.

There are some potential limitations underlying the present study. First, the current experimental design sought to measure participants’ space–time associations along the sagittal and vertical axis, but the sagittal front-back mouse mapping might also conflict with the curved front-right/behind or front-left/behind axis in temporal cognition. In the experiment, each participant held one mouse in front of his/her body, and the second behind his/her body. Nevertheless, participants might not strictly confine themselves to the front-back axis. Rather they may consciously or unconsciously move the back mouse to the right/behind or left/behind side so that they can operate this mouse with a relatively comfortable body position. Consequently, this situation may generate an implicit curved front-right/behind or front-left/behind axis. Any follow-up studies which intend to examine the temporal representations along the sagittal axis should take into consideration how they can to the maximum extent exclude the interference from the curved front-right/behind or front-left/behind axis. A possible solution may be placing both mice in front of the participant, with one closer and one further. Such kind of mouse arrangement can also be viewed as constituting a front-back axis.

Second, given that the pictorial stimuli used in the present study displayed different types of temporal themes, it might be more difficult to make a judgment for some items about “earlier” than “later” or the other way around. These items may accordingly accrue a higher error rate or longer response latency than others. Further studies can do a specific item analysis by comparing participants’ accuracy and RTs on different items so as to examine if the variance among items might affect participants’ performance.

Last but not the least, the design of Experiment 2 did not incorporate a variable, namely, the language context or location of the test (i.e., whether bilinguals were tested in the L1 speaking country or L2 speaking country). Some previous studies indicate that bilinguals behaved like native speakers of the L2 if they were tested in an L2 speaking country (Fuhrman et al., 2011; Chen and O’Seaghdha, 2013). Note that the present research was conducted in mainland China. It remains an open question as to whether ME bilinguals would show temporal thinking patterns analogous to those displayed by native speakers of English when the experiment is carried out in an English-speaking country. Likewise, all the ME bilingual participants in Experiment 2 acquired L2 English in mainland China. Therefore, the findings (i.e., the acquisition of L2 does not affect speakers’ mental representations of temporal sequence) might be limited to bilinguals who acquired L2 in the L1 setting. The question of whether the current conclusion can be generalized to bilinguals who acquired L2 in L2 speaking countries (i.e., those who were immersed in the L2 setting to acquire L2) is pending further examinations.

## Conclusion

*Via* two experiments, we replicate previous findings by showing that English and Mandarin speakers do think about time differently. English speakers access a single sagittal front-to-back MTL, whereas both a sagittal and a vertical top-to-bottom axis are activated in Mandarin speakers’ cognition. This observation is in line with the linguistic relativity hypothesis. However, the present findings also clarify the existing knowledge on the relationship between ME bilingualism and temporal cognition, indicating that ME bilinguals, irrespective of whether they have attained elementary or very advanced level of L2 proficiency, do not reconstruct their mental representations of temporal sequence as compared with Mandarin monolinguals. Several theoretical implications for a more broad research field on bilingualism and cognition can be drawn from the present study, a crucial one being that it provides evidence against the view that L2 acquisition can reshape habitual modes of thinking established by L1. This kind of counter-evidence is clearly shown at least in the conceptual domain of time. Specifically, we highlight the fact that L2 acquisition may not necessarily restructure cognition in some specific domains, even though there are both significant differences and similarities in lexis or grammar between L1 and L2.

## Data Availability Statement

The original contributions presented in the study are included in the article/Supplementary Material, further inquiries can be directed to the corresponding author.

## Ethics Statement

The studies involving human participants were reviewed and approved by Ethics Committee of Yangzhou University. The patients/participants provided their written informed consent to participate in this study. Written informed consent was obtained from the individual(s) for the publication of any potentially identifiable images or data included in this article.

## Author Contributions

WY and YG conducted the research, which was designed together with YF. WY, YG, YF, and YS participated in the data analyses. WY drafted the manuscript. YS helped with editing and commenting. All authors contributed to the article and approved the submitted version.

## Funding

This study was supported by National Social Science Fund of China (16CYY023).

## Conflict of Interest

The authors declare that the research was conducted in the absence of any commercial or financial relationships that could be construed as a potential conflict of interest.

## Publisher’s Note

All claims expressed in this article are solely those of the authors and do not necessarily represent those of their affiliated organizations, or those of the publisher, the editors and the reviewers. Any product that may be evaluated in this article, or claim that may be made by its manufacturer, is not guaranteed or endorsed by the publisher.

## Footnotes

^1^Although Miles et al. (2011) did not specify what the L1/L2 of their ME bilingual participants was, we did discuss with one of the coauthors (L. Tan) of this publication and obtained more cultural and linguistic background information of the participants. These bilingual participants were ethnic Chinese of Singaporean nationalities, and their L1 were indeed Mandarin and L2 English (L. Tan, personal communication, March 20, 2015).

^2^Prior to conducting ANOVAs, tests for homogeneity of variance were carried out. We also performed tests of normality to examine if the data were normally distributed. Results showed that there was homogeneity of variance for all ANOVAs in both Experiment 1 and Experiment 2 (all *p* > .05). Test of normality for dependent variables, i.e., by-participants RTs and by-items RTs on the FE, BE, TE and DE mouse-mapping conditions, respectively, in both experiments, show that all data were normally distributed (*p* > .05), with the exception of by-participants RTs on the BE (*p* = .038) and DE (*p* = .045) conditions by ME bilinguals of advanced English proficiency in Experiment 2. Since the two exceptions did not constitute a strong violation of normal distribution of data, as suggested by the values of *p*, we did not do transformations for these two groups of data.

^3^This is a mock examination simulating the formal IELTS (International English Language Testing System) which is designed to test people’s L2 English proficiency. The paper test includes three subsections, i.e., listening, reading and writing, with a band score ranging from 1 to 9. All the testing items in our mock IELTS are taken from the official mock IELTS websites: https://www.ielts.com/prepare/mock-test.
